# Deep-Learning to Predict BRCA Mutation and Survival from Digital H&E Slides of Epithelial Ovarian Cancer

**DOI:** 10.3390/ijms231911326

**Published:** 2022-09-26

**Authors:** Camilla Nero, Luca Boldrini, Jacopo Lenkowicz, Maria Teresa Giudice, Alessia Piermattei, Frediano Inzani, Tina Pasciuto, Angelo Minucci, Anna Fagotti, Gianfranco Zannoni, Vincenzo Valentini, Giovanni Scambia

**Affiliations:** 1 Fondazione Policlinico Agostino Gemelli, IRCCS, Gynecology and Obstetrics, 00168 Rome, Italy; 2 Fondazione Policlinico Agostino Gemelli, IRCCS, Radiomics Core Facility, 00168 Rome, Italy; 3 Fondazione Policlinico Agostino Gemelli, IRCCS, Pathology, 00168 Rome, Italy; 4 Fondazione Policlinico Agostino Gemelli, IRCCS, Data Collection Core Facility, 00168 Rome, Italy; 5 Fondazione Policlinico Agostino Gemelli, IRCCS, Genomics Core Facility, 00168 Rome, Italy; 6 Fondazione Policlinico Agostino Gemelli, IRCCS, Radiation Oncology, 00168 Rome, Italy

**Keywords:** ovarian cancer, somatic BRCA mutational status, digital pathology, machine learning, artificial intelligence

## Abstract

BRCA 1/2 genes mutation status can already determine the therapeutic algorithm of high grade serous ovarian cancer patients. Nevertheless, its assessment is not sufficient to identify all patients with genomic instability, since BRCA 1/2 mutations are only the most well-known mechanisms of homologous recombination deficiency (HR-d) pathway, and patients displaying HR-d behave similarly to BRCA mutated patients. HRd assessment can be challenging and is progressively overcoming BRCA testing not only for prognostic information but more importantly for drugs prescriptions. However, HR testing is not already integrated in clinical practice, it is quite expensive and it is not refundable in many countries. Selecting patients who are more likely to benefit from this assessment (BRCA 1/2 WT patients) at an early stage of the diagnostic process, would allow an optimization of genomic profiling resources. In this study, we sought to explore whether somatic BRCA1/2 genes status can be predicted using computational pathology from standard hematoxylin and eosin histology. In detail, we adopted a publicly available, deep-learning-based weakly supervised method that uses attention-based learning to automatically identify sub regions of high diagnostic value to accurately classify the whole slide (CLAM). The same model was also tested for progression free survival (PFS) prediction. The model was tested on a cohort of 664 (training set: *n* = 464, testing set: *n* = 132) ovarian cancer patients, of whom 233 (35.1%) had a somatic BRCA 1/2 mutation. An area under the curve of 0.7 and 0.55 was achieved in the training and testing set respectively. The model was then further refined by manually identifying areas of interest in half of the cases. 198 images were used for training (126/72) and 87 images for validation (55/32). The model reached a zero classification error on the training set, but the performance was 0.59 in terms of validation ROC AUC, with a 0.57 validation accuracy. Finally, when applied to predict PFS, the model achieved an AUC of 0.71, with a negative predictive value of 0.69, and a positive predictive value of 0.75. Based on these analyses, we have planned further steps of development such as proving a reference classification performance, exploring the hyperparameters space for training optimization, eventually tweaking the learning algorithms and the neural networks architecture for better suiting this specific task. These actions may allow the model to improve performances for all the considered outcomes.

## 1. Introduction

Epithelial ovarian cancer (EOC) is strongly dominated by copy number changes without a focal gene driving mutation. Approximately half of cases exhibits defective DNA repair via homologous recombination (HR), frequently caused by inactivation of the breast cancer susceptibility (BRCA) genes (overall up to 30%) [1,2,3,4].

Defective HR (HR-d) reflects underlying genomic instability, which has significant therapeutic implications in EOC. It is in fact associated to a striking platinum sensitivity and can be targeted by poly-ADP ribose polymerase inhibitors (PARPi).

Although HR assessment is progressively overcoming BRCA testing, it is not yet integrated in clinical practice and can be challenging and expensive, being also still not refundable in many countries. Selecting patients who are more likely to benefit from it at an early stage of the diagnostic process (somatic BRCA 1/2 wild type [WT] patients), could allow an optimization of genomic profiling resources.

Histologic phenotypes have been recognized to somehow reflect genetic alterations in cancer tissues [5,6,7,8,9,10]. BRCA-mutated (BRCA-mut) EOC were found to have more frequent Solid, pseudo-Endometrioid, and Transitional cell carcinoma-like morphology (SET features) and higher mitotic indexes compared to BRCA WT EOC [11].

Since hematoxylin and eosin (H&E)-stained tissue slides are ubiquitously available for cancer patients, predicting mutations from tissue slides could be a time- and cost-effective method to characterize patients and address them to genomic profile.

Advances in digital pathology and artificial intelligence have presented the potential to analyze gigapixel whole-slide images (WSI), providing information on tissue microenvironment, integrative image-omic, resistence to treatments but also somatic genomic status straight away [12,13,14,15,16,17,18,19,20,21,22,23,24,25,26,27,28,29,30,31,32,33].

WSI is a complex domain with several unique challenges, that requires different deep-learning-based computational pathology approaches such as manual annotation of gigapixel WSIs in fully supervised settings or large datasets with slide-level labels in weakly supervised ones.

The delineation of a pixel, patch or region-of-interest (ROI)-level annotations has produced promising results even if it cannot be directly generalized, and if it may suffer from noisy training labels and reduced reproducibility on data from different sources and imaging devices [34,35,36,37].

On the other hand, weakly supervised approaches demonstrated an exceptional clinical-grade performance [38] but require thousands of WSIs to achieve performance comparable to fully supervised and ROI-level classifiers and may not be suitable for multi-class subtyping problems.

A recent paper proposed a clustering-constrained-attention multiple-instance learning (CLAM) as a high-throughput deep-learning framework that aims to address the key challenges outlined above [39].

The authors demonstrated that such approach can be used to localize well-known morphological features on WSIs without the need for spatial labels, overperforming standard weakly supervised classification algorithms and resulting adaptable to independent test cohorts, smartphone microscopy and different tissue content.

In this study, we aimed at identifying somatic BRCA 1/2 mutational status directly from H&E slides using a CLAM-based approach. In particular we planned to evaluate negative and positive predictive values of the CLAM-based model compared to genomic sequencing as the reference standard. As secondary endpoints, we aimed at evaluating CLAM-based model accuracy in prognosis prediction measured as progression free survival (PFS).

## 2. Results

From November 2016 to November 2020, 1265 consecutive patients underwent BRCA 1/2 testing in our institution.

**Figure 1:**
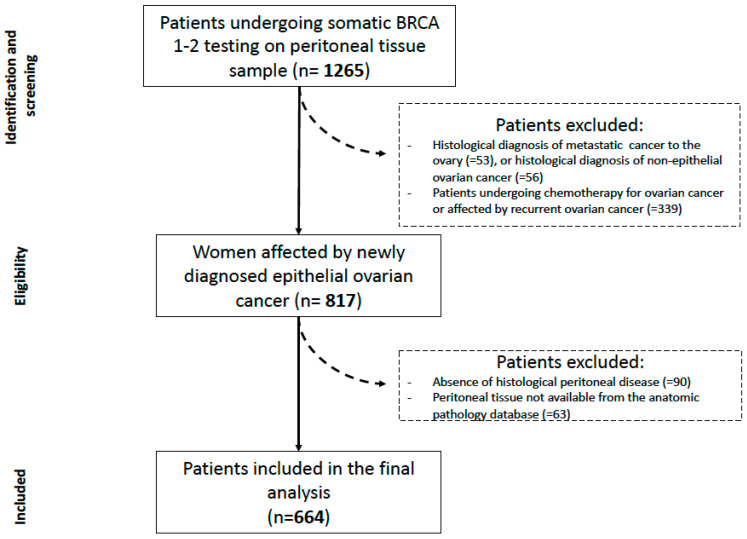
Flowchart of the study.

A total of 664 patients was finally analyzed in the current study (see Figure 1). Regarding secondary endpoints, 8 patients were lost at follow-up and 656 patients were therefore lastly included.

Table 1 shows main clinic-pathological characteristics. Overall, median age of included patients was 61 years old. More than half of the patients had positive family history for cancers (mainly breast). The vast majority of the population had a serous histotype (95.9%), grade 3 (97.1%) and III or IV FIGO stage (92.4%).

Regarding BRCA 1/2 status, 431 (64.9%) patients resulted WT while 233 (35.1%) mutated, 51.5% of which were BRCA 1 mutated. We have reported specific mutations analyzed in the Table 1; with a majority of frameshift mutations of 40.4%.

All mutated patients were addressed to genetic counselling and 86.6% were tested for germline BRCA 1/2 pathogenetic variants. A third (38.2%) had a germline alteration.

Table 2 shows treatment and oncological outcome data. Regarding therapeutic choices, 54.5% of these patients underwent neoadjuvant chemotherapy with a median number of cycles prior to interval debulking surgery of 4, after a laparoscopic assessment. Three hundred and forty out of 644 (52.8%) recurred, but only 27.7% died.

**Figure 2:**
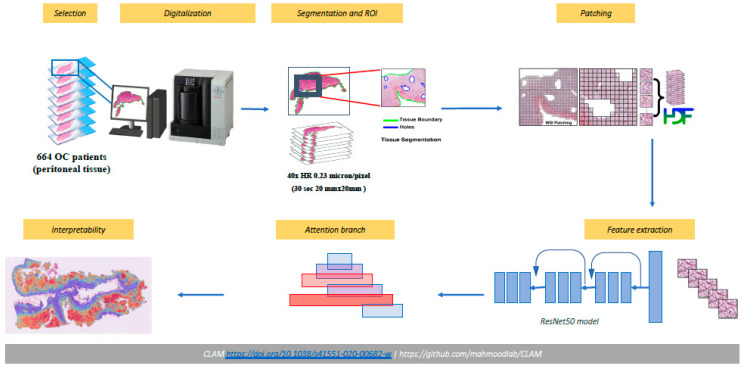
Pipeline of analysis.

The whole process is represented in Figure 2. The outcome was BRCA 1/2 mutated yes/no, in which VUS were considered as mutated patients.

### 2.1. Phase 0: Reference Standard for BRCA Status Prediction

A reference classification performance was established according to the classification accuracy and AUC ROC of an expert pathologist, based on available criteria [11]. On the whole 664 slides of the dataset, the reference performance was as follows: accuracy 0.629, specificity 0.879, sensitivity 0.167, negative predictive value 0.661, positive predictive value 0.428 (TN: 379, TP: 39, FN: 194, FP: 52).

### 2.2. Phase 1: WSI for Somatic BRCA Status Prediction

The dataset was randomly split into into a development set, consisting of a training set and internal validation set, and an hold-out testing. The proportion between development and testing set set was 80% to 20%. Thus, we used 464 images for training (244/220), 132 images for validation (86/46), and 68 images were hold out for testing (44/24).

The performance on the training set was 0.7 in terms of AUC, but on the testing set the AUC was 0.55. In detail, for training set class zero (BRCA wild type) the model correctly identified 153 out of 244 images, while for class one (BRCA mutated) 139 out of 220. In the testing set class zero, the model correctly identified 49 out of 86, while in class one 24 out of 46.

### 2.3. Phase 2: ROI on WSI for Somatic BRCA Status Prediction

For this analysis, a subset of images were used, because of the time consuming process of manual ROI delineation. The process of dataset splitting was the same as before, only with slightly different proportions, so that we used 198 images for training (126/72), 87 images for validation (55/32), and only three images were hold out for testing (2/1), merely to create heatmaps of activation regions on unseen images.

The outcome was BRCA 1/2 mutated yes or no, and VUS were considered as mutated patients.

**Figure 3:**
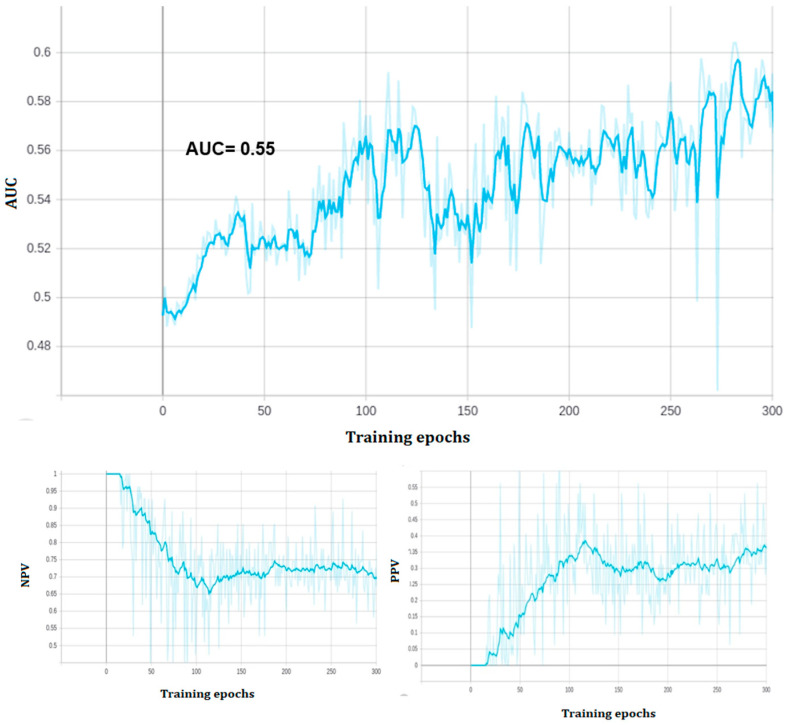
Phase1: Performance of the model during training (300 epochs). Top: validation AUC ROC. Low left: negative predictive value (NPV). Low right: positive predictive value (PPV).

**Figure 4:**
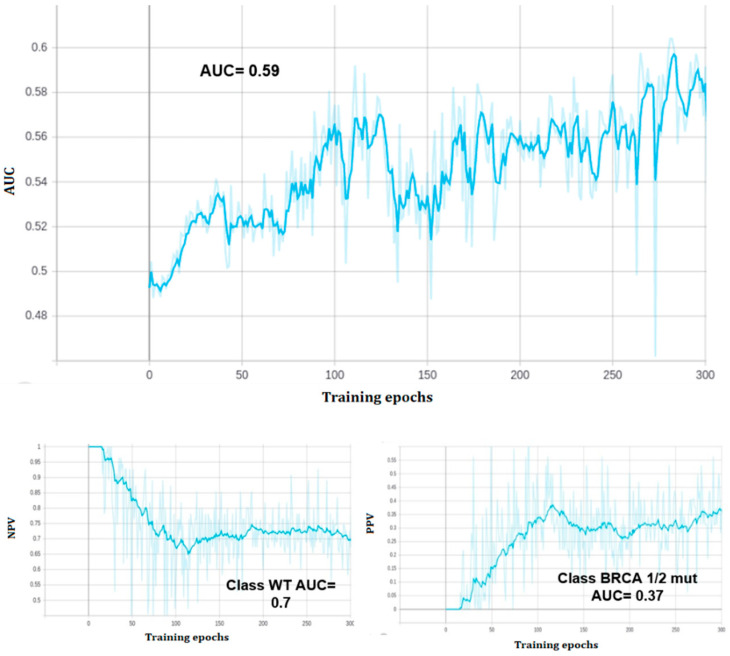
Phase2: Performance of the model during training (300 epochs). Top: validation AUC ROC. Low left: negative predictive value (NPV). Low right: positive predictive value (PPV).

At the end of training, the model reached a zero classification error on the training set, but the performance of the predictive model on the validation set was 0.59 AUC ROC, with 0.57 validation accuracy (see Figure 3 and Figure 4). The model correctly classified 39 out of 55 class zero (BRCA wild type) images, and 11 out of 32 class one (BRCA mutated) images on the validation set. All of the three held out images were correctly classified.

In particular, the model assigned to the BRCA 1/2 mutated held out image a probability of 98% of mutation, while a probability of 38% and <1% were assigned to the other two held out images (both WT), respectively.

### 2.4. Phase 3: Exploration of the Hyperparameters Space for Training Optimization

Given the results obtained in the previous two phases, we looked for chances of performance improvement by exploring the CLAM model hyperparameters space through a grid search. To do so, we let the following hyperparameters vary: patch level between zero and 2; attention branch single or multiple; bag loss function and clustering loss function one between support vector machine or cross entropy; the relative weight of the two loss in the overall loss function between 0.2 and 0.8 with 0.1 steps; the number of highest and lowest attention patches to be fed to the clustering algorithm within the set 4, 8, 16, 32, 64, 100, 500, 1000. The dataset splitting was the same as phase 1. Grid search type was random search with an early stopping criterion on validation loss decreasing. For each patch level, the best five experiments in terms of validation AUC were retained for performance assessment on the testing set. None of these hyperparameters combination led to significant or even relevant BRCA classification performance improvement neither in the validation nor in the testing set.

### 2.5. Phase 4: WSI for Predicting Relapse

For this analysis, slide resolution was taken to be fixed at the highest available value on the image (called “patch level zero” in CLAM framework). As in the previous steps, the dataset was split into a development set (training and validation) and a testing set. Grid search on hyperparameters was performed on the development set to select the five highest performing models, which were later assessed on the hold-out testing set for predictive performance.

The combination of parameters led to a grid search on 64 different models for 200 epochs training length with an early stopping criterion of 20 epochs non-decreasing loss for each outcome.

**Figure 5:**
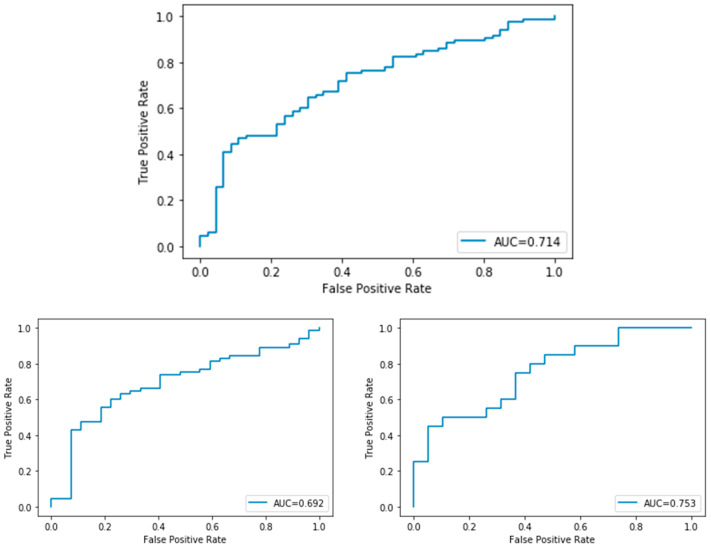
Phase 4: classification on testing set with the highest test AUC model. Top: validation AUC ROC. Bottom left: ROC curve on BRCA mut cases. Bottom right: ROC curves on BRCA WT cases.

On a total of 656 images (229 class 0; 427 class 1), 394 images were assigned to training set, 131 images to validation set, 131 images were hold out for testing (46/85). The AUC on the testing set was 0.71 (see Figure 5).

## 3. Discussion

Molecular profiling in cancer patients has been increasingly important to determine the optimal therapeutic strategy. The combination of the digitization of pathology WSI with deep learning to predict somatic mutations, could be a promising approach to achieve a time- and cost-effective complementary method for personalized treatment.

When applied on our dataset, the available morphological criteria (SET features) showed disappointing results (accuracy 0.629, negative predictive value 0.661). This suggests that phenotype and genotype may not be strongly related, as previously suggested [11].

**Figure 6:**
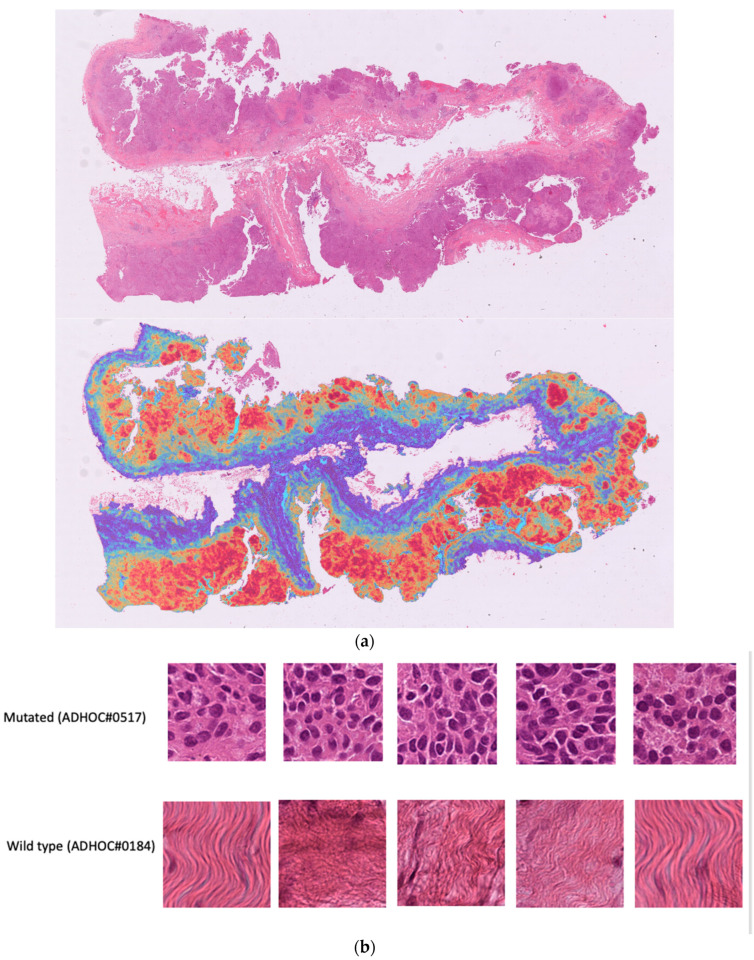

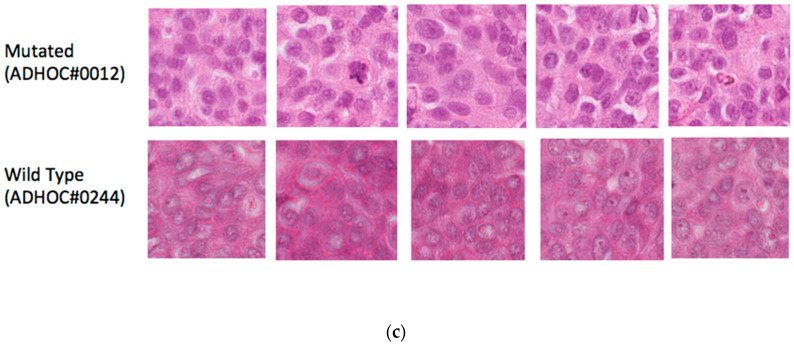
(**a**). Heatmap of the held-out BRCA mutated image. Red area represent areas with higher model activation, i.e., areas where the model recognizes pattern associated to the somatic BRCA mutation. (**b**). Patch level top 5 highest attention patterns (WSI). (**c**). Patch level top 5 highest attention patterns (ROI on WSI).

In our phase 1 (high testing and validation errors), we focused on the specific features/patterns within the tissue images that the model recognized to make response predictions. Looking at the activation map (heatmap) of the highest and lowest prediction on the testing set (see Figure 6a–c), we found that the model identified tumor cells in the mutated cases and stroma in the wild type case which could reflect the morphological differences previously described namely solid phenotype and higher mitotic index [11].

However, given the performances, we hypothesize that the highest attention pattern should be focused on tumor areas on which reported differences might be more evident thus useful for outcome prediction. It was necessary to tweak parameters, starting from optimal tissue identification in the segmentation process.

Unfortunately, the process of manually delineating ROI by a dedicated pathologist did not improve the overall performances and neither did the exploration of the hyperparameters space for training optimization, even tweaking the learning algorithms and the neural networks architecture for better suiting the task of BRCA 1/2 status identification.

Several issues and limitations have been encountered during the analysis.

First, the retrospective nature of the study represents an unavoidable source of selection bias and imaging data inhomogeneity.

Although we collected only H&E slices of peritoneal tissue and checked for a minimum percentage of tumor cells in all specimens, patients were not divided in subgroups according to the type of surgery performed; thus small biopsies obtained from exploratory laparoscopy might have provided less representative specimens and images of lower quality compared to peritonectomies.

Second, the absence of an external validation does not allow us to draw any definitive conclusion on the replicability of the model, though the use of H&E slides for cancer diagnosis is spread all over the world. Moreover, we are well aware that there are concerns of between-center variation in imaging results which might significantly impact on the reproducibility of the model results.

Third, our BRCA 1/2 testing was mainly performed on fresh frozen ovarian cancer tissue. We assumed that all other areas of the disease within the same patient shared the same mutational status but this consideration may not be entirely correct. given the significant EOC heterogeneity.

Fourth, our patients were only screened for BRCA 1/2: no other genes involved in the HRD pathways were included in the analysis. Therefore, we cannot exclude the presence of mutations in the other genes whose mutational status could correlate with imaging features typical of mutated patients. Moreover, any type of BRCA 1/2 pathogenic variant was labeled as “mutated”. There are not enough data to establish whether different mutations produce different downstream effects but we cannot rule out differences in phenotype. This might have affected our analysis.

Fifth, the analysis was carried out using open source pipelines such as the CLAM model which are not customized for the purpose of the study. Entirely in-house designed pipelines, tailored on genomic status identification, might improve final results.

Overall, the model could provide a critical information at the very beginning of the diagnostic process and, if proven effective, tailor further genomic testing (e.g., only BRCA testing or HRD testing) and optimizing genomic testing resources.

### Our Results in the Context of Other Observations

Preliminary but encouraging results have been published in the last 3 years on computational pathology.

In 2020, Jang and colleagues showed that APC, KRAS, PIK3CA, SMAD4, and TP53 mutations can be predicted from H&E pathology images using deep learning-based classifiers [40]. The AUCs for ROC curves ranged from 0.693 to 0.809 for frozen WSIs and from 0.645 to 0.783 for the FFPE WSIs.

Xu et al., developed a deep learning model to accurately classify chromosomal instability status on a cohort of 1010 patients with breast cancer (Training set: *n* = 858, Test set: *n* = 152) from The Cancer Genome Atlas achieving, an area under the curve of 0.822 with 81.2% sensitivity and 68.7% specificity in the test set. Patch-level predictions of chromosomal instability status suggested intra-tumor heterogeneity within slides [41].

Fu et al. in 2020 quantified histopathological patterns across 17,396 H&E stained histopathology slide images from 28 cancer types and successfully correlate these with matched genomic, transcriptomic and survival data [5].

Moreover, computational histopathology highlighted prognostically relevant areas, such as necrosis or lymphocytic aggregates. The authors underlined the remarkable potential of computer vision in characterizing the molecular basis of tumor histopathology.

Kiehl et al. developed a deep learning model from routine histological slides and/or clinical data to predict lymph node metastasis in colorectal cancer [42]. The deep learning model achieved an AUROC of 71.0%, the clinical classifier achieved an AUROC of 67.0% and a combination of the two classifiers yielded an improvement to 74.1%.

Finally, Wang et al. trained a deep convolutional neural network of ResNet on WSIs to predict the gBRCA mutation in breast cancer [43]. One hundred and sixty six images were combined from two different datasets. The model reached in the external validation dataset an AUC of 0.766 (0.763–0.769) at 40× magnification. The authors reported the role of histological grade on the accuracy of the prediction.

It has also to be acknowledged that new data are emerging regarding the relevance of BRCA status in the upfront surgical treatment. Not only BRCA WT OC patients seems to benefit more that BRCA mutated ones from hyperthermic intraperitoneal chemotherapy performed at primary debulking surgery but even a neoadjuvant chemotherapy approach has been supposed to be less detrimental in patients harboring BRCA mutation [44,45,46]. If these data are confirmed, the turnaround time of BRCA status acquisition will became crucial. Artificial intelligence applied to digital pathology holds much promise in bringing innovative solutions to this possible future clinical unmet need.

## 4. Materials and Methods

### 4.1. Patients and Study Design

This is an observational study with patients retrospectively enrolled at the Fondazione Policlinico Universitario “Agostino Gemelli” IRCCS of Rome, Italy, from November 2016 to November 2020.

A weakly supervised deep learning-based model on H&E in EOC patients was set up for BRCA1/2 status prediction.

The retrospective data on BRCA1/2 testing performed on patients with NGS technique was considered as the reference standard of the computational pathology analysis. H&E slides were prepared by a technician and evaluated by a dedicated pathologist, according to current international indications.

In a second step, clinical and follow-up data including therapeutic regimens, progression free survival (PFS) and overall survival (OS), were considered as outcomes to be predicted.

This study was conducted in accordance with the declaration of Helsinki and was approved by the Ethical committee of Fondazione Policlinico Universitario Agostino Gemelli IRCCS (Prot.; 001134″3/21; ID: 3894, 25 March 2021), with the requirement for informed consent. The research was founded by the Italian Ministry of Health providing Institutional Financial Support 5 × 1000 (2020).

### 4.2. Study Population

Eligible population includes: (i) women affected by EOC, with known somatic BRCA 1/2 mutational profile and (ii) available Formalin-Fixed Paraffin-Embedded peritoneal tissue sample, collected at the time of first diagnosis of EOC with at least 30% of cancer cells.

For the second step only those patients for whom we had complete follow-up information (minimum follow up 18 months) were included.

The exclusion criteria were: (i) patients affected by recurrent ovarian cancer; (ii) samples collected after chemotherapy; (iii) patients with extra-ovarian tumors with metastases to ovaries; (iv) patients with history of other malignancies in the past 5 years; (v) patients who received any type of target therapy prior to EOC diagnosis.

All the enrolled women were required to sign written informed consent.

Standardized procedures according to previously published workflows were observed to achieve somatic BRCA 1/2 genes mutational status [47,48,49].

### 4.3. Deep Learning Approach (CLAM)

CLAM is a deep-learning-based weakly supervised method that uses attention-based learning to automatically identify sub regions of high diagnostic value to accurately classify the whole slide, while also enabling the use of instance-level clustering over the representative regions identified to constrain and refine the feature space.

CLAM is publicly available as a Python package over GitHub (https://github.com/mahmoodlab/CLAM, accessed on 29 August 2022) [50].

For whole-slide-level learning without annotation, CLAM uses an attention-based pooling function [51] to aggregate patch-level features into slide-level representations for classification. At a high level, during both training and inference, the model examines and ranks all patches in the tissue regions of a WSI, assigning an attention score to each patch, which informs its contribution or importance to the collective slide-level representation for a specific class.

This interpretation of the attention score is reflected in the slide-level aggregation rule of attention-based pooling, which computes the slide-level representation as the average of all patches in the slide weighted by their respective attention score. Unlike the standard MIL algorithm [45,46], which was designed and widely used for weakly supervised positive/negative binary classification (for example, cancer versus normal), CLAM is designed to solve generic multi-class classification problems. A CLAM model has N parallel attention branches that together calculate N unique slide-level representations, where each representation is determined from a different set of highly attended regions in the image viewed by the network as strong positive evidence for the one of N classes in a multi-class diagnostic task. Each class-specific slide representation is then examined by a classification layer to obtain the final probability score predictions for the whole slide.

The slide-level ground-truth label and the attention scores predicted by the network can be used to generate pseudo labels for both highly and weakly attended patches as a technique to increase the supervisory signals for learning a separable patch-level feature space. During training, the network learns from an additional supervised learning task of separating the most- and least-attended patches of each class into distinct clusters. In addition, it is possible to incorporate domain knowledge into the instance-level clustering to add further supervision.

The pipeline provided by Lu et al., first automatically segments the tissue region of each slide and divides it into many smaller patches, so that they can serve as direct inputs to a CNN. Next, using a CNN for feature extraction, the tool converts all tissue patches into sets of low-dimensional feature embeddings. Following this feature extraction, both training and inference can occur in the low-dimensional feature space instead of the high-dimensional pixel space. The volume of the data space is decreased nearly 200-fold, drastically reducing the subsequent computation required to train supervised deep-learning models.

### 4.4. WSI Processing

#### 4.4.1. Scanning

Clinical slides were reviewed with the supervision of a dedicated pathologist and selected hematoxylin and eosin-stained slides containing tumor were scanned using the C13220-31 NanoZoomer S360 (Hamamatsu, Japan).

Each slide was scanned using a 40× objective lens (scanning resolution 0.23 µM/pixel) of the NanoZoomer and the slide code details, the scanning area and the number of focus points for each slide were determined by the user. The number of focal points was approximately 15 focus points per slide.

Place the glass slides in the cassettes and set them in the holder of the machine, each slide was automatically handled and scanned. The approximate time taken to scan the image of the whole slide per case was up to 1 min.

Scanned images in their proprietary NDP Image (NDPI) file format were checked for the whole and details of the tissues using the NDP.view2 image viewing software for NanoZoomer on a desktop computer with a high-definition resolution screen (1920 × 1080 pixels). NDPI stores an image pyramid in TIFF directory entries.

Images and data were stored and exported to an external storage device.

#### 4.4.2. Segmentation

The first step is an automated segmentation of the tissue regions. The first step focuses on segmenting the tissue and excluding any holes. The segmentation of specific slides can be adjusted by tuning the individual parameters. The pipeline input is digitized whole slide image data in well-known standard formats (.svs, .ndpi, .tiff etc.). The WSI is read into memory at a down sampled resolution, converted from RGB to the HSV color space. A binary mask for the tissue regions (foreground) is computed based on thresholding the saturation channel of the image after median blurring to smooth the edges and is followed by additional morphological closing to fill small gaps and holes [52]. The approximate contours of the detected foreground objects are then filtered based on an area threshold and stored for downstream processing while the segmentation mask for each slide is made available for optional visual inspection. A human-readable text-file is also automatically generated, which includes the list of files processed along with editable fields containing the set of key segmentation parameters used.

#### 4.4.3. Patching

After segmentation, the background is removed from images for each slide and the remaining pixels are grouped into a grid of smaller images (256 × 256 patches) from within the segmented foreground contours at the user-specified magnification and stores stacks of image patches along with their coordinates and the slide metadata using the Hierarchical Data Format version 5 (HDF5).

This is not a computationally intensive process and is dependent on resolution level.

The number of patches extracted from each slide can range from hundreds (biopsy slide patched at ×20 magnification) to hundreds of thousands (large resection slide patched at ×40 magnification). Output is a representation of images through patches in a high dimensional feature (HDF) space generated by a pre-trained convolutional neural network.

#### 4.4.4. Feature Extraction

Following patching, we used the pre-trained ResNet50 model [53] already embedded in CLAM to compute a low-dimensional feature representation for each image patch of each slide. Features extraction from patches is a computationally intensive step. Requiring about one minute per whole slide image on NVIDIA Quadro RTX 5000.

#### 4.4.5. Attention Branch

Each patch feature vector then enters an attention network which is trained to recognize patterns often associated with a particular Slide-level label (over-simplification).

At the end of training, the overall model should be able to identify characteristic regions of activation and make classification at the whole slide image level.

### 4.5. Statistical Analysis

The sample size available for the analysis consisted of 664 histological images. For computational reasons, the pipeline was first applied to about 30% of available data (171 images) and then incremented to 298 in order to measure the performance gain due to increased sample size.

The dataset was split into a training and validation set, respectively 80% and 20% of the considered number of samples.

Classification performance of the predictive model was monitored during training with ROC AUC, negative and positive predictive value on the validation set. The final model was then applied to held out images to generate activation maps on unseen BRCA mutated images. The model was trained for 300 epochs on a NVDIA Quadro RTX 5000. All statistical analysis was performed in Python version 3.7.4.

## 5. Conclusions

Our results confirm that models applied to H&E slides cannot yet match the performance level of the gold standards thus their use in current clinical practice cannot be advocated. Nevertheless, its potentiality as a screening tool for personalization and optimization of genomic testing warrants further investigation.

From a clinical point of view, the information obtained directly from frozen section H&E slides, may give clinicians a crucial information at an early stage of the therapeutic decision making process that could be integrated with already validated clinical scores or multiomics translational approaches [54,55,56].

For all these reasons, we believe that further developments are well worth carrying out. We have already planned to enlarge the dataset collecting cases from December 2020 to August 2022. Moreover, we are working on improving data input for CLAM analysis by using a recently released segmentation model code (https://github.com/MSKCC-Computational-Pathology/DMMN-ovary, accessed on 29 August 2022) [57]. On the other hand, we are exploring in collaboration with other groups, new approaches such as the development of a persistent homology-based model on the same dataset [58]. 

Future studies must include data across multiple centers not used in the model training to demonstrate high accuracy and reproducibility. A deeper involvement of pathologists should be pursued in order to achieve the finest tuning possible according to well recognized features. 

## Figures and Tables

**Table 1:** Clinical, pathological and surgical characteristics of the study population.

Characteristic	All Cases *n* = 664
Age	
Mean ± SD	60.6 ± 12.1
Median (min-max)	61 (23–87)
BMI *	
Mean ± SD	25.2 ± 5.5
Median (min-max)	24.4 (−1–55.6)
Familiarity	461 (69.4)
Type of tumor	
Mammary	182/461 (39.5)
Ovarian	64/461 (13.9)
Prostate	31/461 (6.7)
Gastrointestinal	121/461 (26.2)
Other	292/461 (63.3)
Histotype	
Serous Carcinoma	637 (95.9)
Mucinous Carcinoma	1 (0.2)
Clear Cells Carcinoma	5 (0.8)
Endometroid Carcinoma	8 (1.2)
Other	13 (2)
Grading	
1	6/661 (0.9)
2	8/661 (1.2)
3	642/661 (97.1)
Not applicable	5/661 (0.8)
FIGO Stage	
IIB	14 (2.1)
IIIA1	6 (0.9)
IIIA2	4 (0.6)
IIIB	26 (3.9)
IIIC	390 (58.7)
IVA	38 (5.7)
IVB	186 (28)
Somatic BRCA	
Wild type	431 (64.9)
Mutated	233 (35.1)
BRCA 1	120/233 (51.5)
BRCA 2	71/233 (30.5)
BRCA 1-2	1/233 (0.4)
VUS BRCA 1	19/233 (8.2)
VUS BRCA 2	22/233 (9.4)
Germline BRCA	
Wild type	118/202 (58.4)
BRCA 1	48/202 (23.8)
BRCA2	29/202 (14.4)
BRCA 1 and BRCA 2	0/202 (0)
VUS BRCA 1 VUS BRCA 2	5/202 (2.5) 2/202 (1)
Type of mutation variant	
Frameshift mutation	94/233 (40.4)
BRCA 1	56/94 (59.6)
BRCA 2	37/94 (39.5)
BRCA 1-2	1/94 (0.01)
Missense mutation	45/233 (19.4)
BRCA 1	7/45 (15.5)
BRCA 2	5/45 (11.1)
VUS BRCA 1	16/45 (35.6)
VUS BRCA 2	17/45 (37.8)
Nonsense mutation	60/233 (25.8)
BRCA 1	39/60 (65)
BRCA 2	20/60 (33.3)
VUS BRCA 2	1/60 (1.7)
Splicing mutation	15/233 (6.4)
BRCA 1	5/15 (33.3)
BRCA 2	5/15 (33.3)
VUS BRCA 1	2/15 (13.4)
VUS BRCA 2	3/15 (20)
Mutation in Copy Number Variation	14/233 (6)
BRCA 1	12/14 (85.7)
BRCA 2	2/14 (14.3)
Splicing/Missense mutation	2/233 (0.8)
BRCA 2	2/2 (100)
Mutation in 3′ UTR	1/233 (0.4)
VUS BRCA 2	1/1 (100)
Intronic mutation	1/233 (0.4)
VUS BRCA 1	1/1 (100)
Synonimous mutation	1/233 (0.4)
VUS BRCA 1	1/1 (100)
Type of surgery	
PDS	294 (44.3)
Diagnostic	370 (55.8)
Further surgery	
None	88/664 (13.3)
Attempt at IDS	28/664 (4.2)
IDS	245/664 (36.9)
Restaging	3/664 (0.5)
Other	6/664 (0.9)
LPS PIV at diagnosis §	8 (0–14)
Residual tumor at debulking	
0	254/294 (86.4)
≤1 cm	27/294 (9.2)
>1 cm	13/294 (4.4)
CRS	
1	13/55 (23.6)
2	26/55 (47.3)
3	16/55 (29.1)

Results are presented as *n* (%) except where indicated. BMI: Body Mass Index. FIGO: Federation of International of Gynecologists and Obstetricians. BRCA: BReast CAncer gene. VUS: Variants of Uncertain Significance. PDS: Primary Debulking Surgery. IDS: Interval Debulking Surgery. LPS: LaParoScopy. PIV: Peak Integral Value. * Information available for 642/664 patients. § Information available for 649/664 patients.

**Table 2:** Treatment and oncological outcome of the study population.

Characteristic	All Cases *n* = 664
Chemotherapy	
Clinical Setting	
Adjuvant	223/510 (43.7)
Neoadjuvant	278/510 (54.5)
Not Applicable	9/510 (1.8)
Chemotherapy regimen	
Platinum based	454/501 (90.6)
Other	47/501 (9.4)
Bevacizumab	163/164 (99.4)
Number of cycles *	14.8 ± 8.1
Chemotherapy experimental protocol	118/488 (24.2)
Dose frequency	
Thrice-weekly	392/485 (80.8)
Weekly	85/485 (17.5)
Quatri-weekly	8/485 (1.6)
Number of pre-IDS cycles §	4 ± 1.5
Number of post-IDS cycles †	2.5 ± 1.0
Total number of cycles ‡	6.1 ± 1.8
Time to chemotherapy ¶	105 ± 1481.8
Time to chemotherapy post-IDS ¥	39.8 ± 13.9
Toxicity	204/439 (46.5)
Change of chemotherapy regimen during treatment	43/458 (9.4)
Post-CHT Ca125 Ω	130 ± 585.6
Recist Response	
Complete	60/145 (41.4)
Partial (at least 30% reduction)	49/145 (33.8)
Stability	20/145 (13.8)
Progression (at least 20% increase)	16/145 (11.0)
Serological Response (GCIG)	
Complete	190/268 (70.9)
Partial	63/268 (23.5)
Stable	7/268 (2.6)
Progression	8/268 (3.0)
Relapse/Progression Yes No	427/656 (65.1) 229/656 (34.9)
Death	179/647 (27.7)
Status	
Alive	463/647 (71.6)
Dead for the Disease	172/647 (26.6)
Dead for other causes	7/647 (1.1)
Lost at Follow Up	5/647 (0.8)
Overall survival Ψ	24.7 ± 14.8
PFI	
<6 Months	109/427 (25.5)
6–12 Months	126/427 (29.5)
>12 Months	192/427 (45.0)

Results are presented as *n* (%) except where indicated. IDS: Interval Debulking Surgery. GCIG: Gynecologic Cancer InterGroup. PFI: Platinum-Free Interval. * Information available for 127/664 patients. § Information available for 243 out of 278 patients. † Information available for 175/278 patients. ‡ Information available for 398/664 patients. ¶ Information available for 494/664 patients. ¥ Information available for 184/278 patients. Ω Information available for 359/664 patients. Ψ Information available for 427/664 patients.

## References

[1] Konstantinopoulos P.A., Ceccaldi R., Shapiro G.I., D’Andrea A.D. (2015). Homologous Recombination Deficiency: Exploiting the Fundamental Vulnerability of Ovarian Cancer. Cancer Discov..

[2] Alsop K., Fereday S., Meldrum C., deFazio A., Emmanuel C., George J., Dobrovic A., Birrer M.J., Webb P.M., Stewart C. (2012). BRCA mutation frequency and patterns of treatment response in BRCA mutation-positive women with ovarian cancer: A report from the Australian Ovarian Cancer Study Group. J. Clin. Oncol. Off. J. Am. Soc. Clin. Oncol..

[3] Pal T., Permuth-Wey J., Betts J.A., Krischer J.P., Fiorica J., Arango H., LaPolla J., Hoffman M., Martino M.A., Wakeley K. (2005). BRCA1 and BRCA2 mutations account for a large proportion of ovarian carcinoma cases. Cancer.

[4] Hennessy B.T., Timms K.M., Carey M.S., Gutin A., Meyer L.A., Flake II D.D., Abkevich V., Potter J., Pruss D., Glenn P. (2010). Somatic mutations in BRCA1 and BRCA2 could expand the number of patients that benefit from poly (ADP ribose) polymerase inhibitors in ovarian cancer. J. Clin. Oncol. Off. J. Am. Soc. Clin. Oncol..

[5] Fu Y., Jung A.W., Torne R.V., Gonzalez S., Vöhringer H., Shmatko A., Yates L., Jimenez-Linan M., Moore L., Gerstung M. (2020). Pan-cancer computational histopathology reveals mutations, tumor composition and prognosis. Nat. Cancer.

[6] Ninomiya H., Hiramatsu M., Inamura K., Nomura K., Okui M., Miyoshi T., Okumura S., Satoh Y., Nakagawa K., Nishio M. (2009). Correlation between morphology and EGFR mutations in lung adenocarcinomas: Significance of the micropapillary pattern and the hobnail cell type. Lung Cancer.

[7] Warth A., Penzel R., Lindenmaier H., Brandt R., Stenzinger A., Herpel E., Goeppert B., Thomas M., Herth F.J.F., Dienemann H. (2014). EGFR, KRAS, BRAF and ALK gene alterations in lung adenocarcinomas: Patient outcome, interplay with morphology and immunophenotype. Eur. Respir. J..

[8] Mosquera J.M., Perner S., Demichelis F., Kim R., Hofer M.D., Mertz K.D., Paris P.L., Simko J., Collins C., Bismar T.A. (2007). Morphological features of TMPRSS2-ERG gene fusion prostate cancer. J. Pathol..

[9] Hakimi A.A., Tickoo S.K., Jacobsen A., Sarungbam J., Sfakianos J.P., Sato Y., Morikawa T., Kume H., Fukayama M., Homma Y. (2015). TCEB1-mutated renal cell carcinoma: A distinct genomic and morphological subtype. Mod. Pathol..

[10] Weisman P.S., Ng C.K., Brogi E., Eisenberg R.E., Won H.H., Piscuoglio S., De Filippo M.R., Ioris R., Akram M., Norton L. (2016). Genetic alterations of triple negative breast cancer by targeted next-generation sequencing and correlation with tumor morphology. Mod. Pathol..

[11] Soslow R.A., Han G., Park K.J., Garg K., Olvera N., Spriggs D.R., Kauff N.D., Levine D.A. (2012). Morphologic patterns associated with BRCA1 and BRCA2 genotype in ovarian carcinoma. Mod. Pathol..

[12] Bera K., Schalper K.A., Madabhushi A. (2019). Artificial intelligence in digital pathology-new tools for diagnosis and precision oncology. Nat. Rev. Clin. Oncol..

[13] Niazi M.K.K., Parwani A.V., Gurcan M.N. (2019). Digital pathology and artificial intelligence. Lancet Oncol..

[14] Hollon T.C., Pandian B., Adapa A.R., Urias E., Save A.V., Khalsa S.S.S., Eichberg D.G., D’Amico R.S., Farooq Z.U., Lewis S. (2020). Near real-time intraoperative brain tumor diagnosis using stimulated raman histology and deep neural networks. Nat. Med..

[15] Kather J.N., Pearson A.T., Halama N., Jager D., Krause J., Loosen S.H., Marx A., Boor P., Tacke F., Neumann U.P. (2019). Deep learning can predict microsatellite instability directly from histology in gastrointestinal cancer. Nat. Med..

[16] Bulten W., Pinckaers H., van Boven H., Vink R., de Bel T., van Ginneken B., van der Laak J., Hulsbergen-van de Kaa C.A., Litjens G. (2020). Automated deep-learning system for gleason grading of prostate cancer using biopsies: A diagnostic study. Lancet Oncol..

[17] Ström P., Kartasalo K., Olsson H., Solorzano L., Delahunt B., Berney D.M., Bostwick D.G., Evans A., Grignon D., Humphrey P.A. (2020). Artificial intelligence for diagnosis and grading of prostate cancer in biopsies: A population-based, diagnostic study. Lancet Oncol..

[18] Schapiro D., Jackson H.W., Raghuraman S., Fischer J.R., Zanotelli V.R.T., Schulz D., Giesen C., Catena R., Varga Z., Bodenmiller B. (2017). HistoCAT: Analysis of cell phenotypes and interactions in multiplex image cytometry data. Nat. Methods.

[19] Moen E., Bannon D., Kudo T., Graf W., Covert M., van Valen M. (2019). Deep learning for cellular image analysis. Nat. Methods.

[20] Mahmood F., Borders D., Chen R., McKay G.N., Salimian K.J., Baras A., Durr N.J. (2019). Deep adversarial training for multi-organ nuclei segmentation in histopathology images. IEEE Trans. Med. Imaging.

[21] Graham S., Dang Vu S., Ahmed Raza S.E., Azam A., Tsang Y.W., Kwak J.T., Rajpoot N. (2019). Hover-net: Simultaneous segmentation and classification of nuclei in multi-tissue histology images. Med. Image Anal..

[22] Saltz J., Gupta R., Hou L., Kurk T., Singh P., Nguyen V., Samaras D., Shroyer K.R., Zhao T., Batiste R. (2018). Spatial organization and molecular correlation of tumor-infiltrating lymphocytes using deep learning on pathology images. Cell Rep..

[23] Javed S., Mahmood A., Fraz M.M., Koohbanani N.A., Benes K., Tsang Y.W., Hewitt K., Epstein D., Snead D., Rajpoot N. (2020). Cellular community detection for tissue phenotyping in colorectal cancer histology images. Med. Image Anal..

[24] Mobadersany P., Yousefia S., Amgada M., Gutmanb D.A., Barnholtz-Sloanc J.S., Velázquez Vega J.E., Brat D.J., Cooper L.A.D. (2018). Predicting cancer outcomes from histology and genomics using convolutional networks. Proc. Natl. Acad. Sci. USA.

[25] Heindl A., Khan A.M., Rodrigues D.N., Eason K., Sadanandam A., Orbegoso Aguilar C., Punta M., Sottoriva A., Lise S., Banerjee S. (2018). Microenvironmental niche divergence shapes brca1-dysregulated ovarian cancer morphological plasticity. Nat. Commun..

[26] Yuan Y., Failmezger H., Rueda O.M., Ali H.R., Gräf S., Chin S.F., Schwarz R.F., Curtis C., Dunning M.J., Bardwell H. (2012). Quantitative image analysis of cellular heterogeneity in breast tumors complements genomic profiling. Sci. Transl. Med..

[27] Lazar A.J., McLellan M.D., Bailey M.H., Miller C.A., Appelbaum E.L., Cordes M.G., Fronick C.C., Fulton L.A., Fulton R.S., Mardis E.R. (2017). Comprehensive and integrated genomic characterization of adult soft tissue sarcomas. Cell.

[28] Kather J.N., Heij L.R., Grabsch H.I., Kooreman L.F.S., Loeffler C., Echle A., Krause J., Muti H.S., Niehues J.M., Sommer K.A.J. (2020). Pan-cancer image-based detection of clinically actionable genetic alterations. Nat. Cancer.

[29] Chen R.J., Lu M.Y., Wang J., Williamson D.F.K., Rodig S.J., Lindeman N.I., Mahmood F. (2020). Pathomic fusion: An integrated framework for fusing histopathology and genomic features for cancer diagnosis and prognosis. IEEE Trans. Med. Imaging.

[30] Beck A.H., Sangoi A.R., Leung S., Marinelli R.J., Nielsen T.O., van de Vijver M.J., West R.B., van de Rijn M., Koller D. (2011). Systematic analysis of breast cancer morphology uncovers stromal features associated with survival. Sci. Transl. Med..

[31] Yamamoto Y., Tsuzuki T., Akatsuka J., Ueki M., Morikawa H., Numata Y., Takahara T., Tsuyuki T., Tsutsumi K., Nakazawa R. (2019). Automated acquisition of explainable knowledge from unannotated histopathology images. Nat. Commun..

[32] Pell R., Oien K., Robinson M., Pitman H., Rajpoot N., Rittscher J., Snead D., Verrill C. (2019). The use of digital pathology and image analysis in clinical trials. J. Pathol. Clin. Res..

[33] Bejnordi B.E., Veta M., van Diest P.J., van Ginneken B., Karssemeijer N., Litjens G., van der Laak J.A.W.M. (2017). Diagnostic assessment of deep learning algorithms for detection of lymph node metastases in women with breast cancer. JAMA.

[34] Chen P.H.C., Gadepalli K., MacDonald R., Liu Y., Kadowaki S., Nagpal K., Kohlberger T., Dean J., Corrado G.S., Hipp J.D. (2019). An augmented reality microscope with real-time artificial intelligence integration for cancer diagnosis. Nat. Med..

[35] Nagpal K., Foote F., Tan F., Liu Y., Chen P.H.C., Steiner D.F., Manoj N., Olson N., Smith J.L., Mohtashamian A. (2019). Development and validation of a deep learning algorithm for improving gleason scoring of prostate cancer. npj Digit. Med..

[36] Wang S., Zhu Y., Yu L., Chen H., Lin H., Wan X., Fan X., Heng P.-A. (2019). RMDL: Recalibrated multi-instance deep learning for whole slide gastric image classification. Med. Image Anal..

[37] Coudray N., Ocampo P.S., Sakellaropoulos T., Navneet N., Matija S., Fenyo D., Moreira A.L., Narges R., Razavian N., Tsirigos A. (2018). Classification and mutation prediction from non-small cell lung cancer histopathology images using deep learning. Nat. Med..

[38] Campanella G., Hanna M.G., Geneslaw L., Miraflor A.P., Werneck Krauss Silva V., Busam K.J., Brogi E., Reuter V., Klimstra D.S., Fuchs T.J. (2019). Clinical-grade computational pathology using weakly supervised deep learning on whole slide images. Nat. Med..

[39] Ming Y.L., Williamson D.F.K., Chen T.Y., Chen R.J., Barbieri M., Mahmood F. (2021). Data-efficient and weakly supervised computational pathology on whole-slide images. Nat. Biomed. Eng..

[40] Jang H.J., Lee A., Kang J., Song I.H., Lee S.H. (2020). Prediction of clinically actionable genetic alterations from colorectal cancer histopathology images using deep learning. World J. Gastroenterol..

[41] Xu Z., Verma A., Naveed U., Bakhoum S.F., Khosravi P., Elemento O. (2021). Deep learning predicts chromosomal instability from histopathology images. iScience.

[42] Kiehl L., Kuntz S., Höhn J., Jutzi T., Krieghoff-Henning E., Kather J.N., Holland-Letz T., Kopp-Schneider A., Chang-Claude J., Brobeil A. (2021). Deep learning can predict lymph node status directly from histology in colorectal cancer. Eur. J. Cancer.

[43] Wang X., Zou C., Zhang Y., Li X., Wang C., Ke F., Chen J., Wang W., Wang D., Xu X. (2021). Prediction of BRCA Gene Mutation in Breast Cancer Based on Deep Learning and Histopathology Images. Front. Genet..

[44] Ghirardi V., De Felice F., D’Indinosante M., Bernardini F., Giudice M.T., Fagotti A., Scambia G. (2022). Hyperthermic intraperitoneal chemotherapy (HIPEC) after primary debulking surgery in advanced epithelial ovarian cancer: Is BRCA mutational status making the difference?. Cancer Treat. Res. Commun..

[45] Petrillo M., Marchetti C., De Leo R., Musella A., Capoluongo E., Paris I., Benedetti Panici P., Scambia G., Fagotti A. (2017). BRCA mutational status, initial disease presentation, and clinical outcome in high-grade serous advanced ovarian cancer: A multicenter study. Am. J. Obstet. Gynecol..

[46] Mahdi H., Gockley A., Esselen K., Marquard J., Nutter B., Yang B., Hinchcliff E., Horowitz N., Rose P.G. (2015). Outcome of neoadjuvant chemotherapy in BRCA1/2 mutation positive women with advanced-stage Müllerian cancer. Gynecol. Oncol..

[47] Marchetti C., Minucci A., D’Indinosante M., Ergasti R., Arcieri M., Capoluongo E.D., Pietragalla A., Caricato C., Scambia G., Fagotti A. (2020). Feasibility of tumor testing for BRCA status in high-grade serous ovarian cancer using fresh-frozen tissue based approach. Gynecol. Oncol..

[48] Costella A., De Leo R., Guarino D., D’Indinosante M., Concolino P., Mazzuccato G., Urbani A., Scambia G., Capoluongo E., Fagotti A. (2018). High-resolution melting analysis coupled with next-generation sequencing as a simple tool for the identification of a novel somatic BRCA2 variant: A case report. Hum. Genome Var..

[49] Concolino P., Rizza R., Mignone F., Costella A., Guarino D., Carboni I., Capoluongo E., Santonocito C., Urbani A., Minucci A. (2018). A comprehensive BRCA1/2 NGS pipeline for an immediate Copy Number Variation (CNV) detection in breast and ovarian cancer molecular diagnosis. Clin. Chim. Acta.

[50] Data-Efficient and Weakly Supervised Computational Pathology on Whole Slide Images. https://github.com/mahmoodlab/CLAM.

[51] Wang F., Jiang M., Qian C., Yang S., Li C., Zhang H., Wang X., Tang X. Residual attention network for image classification. Proceedings of the IEEE Conference on Computer Vision and Pattern Recognition.

[52] Maragos P. (1987). Tutorial on advances in morphological image processing and analysis. Opt. Eng..

[53] He K., Zhang X., Ren S., Sun J. Deep residual learning for image recognition. Proceedings of the IEEE Conference on Computer Vision and Pattern Recognition.

[54] De Maria Marchiano R., Di Sante G., Piro G., Carbone C., Tortora G., Boldrini L., Pietragalla A., Daniele G., Tredicine M., Cesario A. (2021). Translational Research in the Era of Precision Medicine: Where We Are and Where We Will Go. J. Pers. Med..

[55] Fagotti A., Ferrandina G., Fanfani F., Ercoli A., Lorusso D., Rossi M., Scambia G. (2006). A laparoscopy-based score to predict surgical outcome in patients with advanced ovarian carcinoma: A pilot study. Ann. Surg. Oncol..

[56] Vizzielli G., Costantini B., Tortorella L., Pitruzzella I., Gallotta V., Fanfani F., Gueli Alletti S., Cosentino F., Nero C., Scambia G. (2016). A laparoscopic risk-adjusted model to predict major complications after primary debulking surgery in ovarian cancer: A single-institution assessment. Gynecol. Oncol..

[57] Ovarian Cancer Segmentation Using Deep Multi-Magnification Network. https://github.com/MSKCC-Computational-Pathology/DMMN-ovary.

[58] Herbert E., Harer J.L. (2010). Computational Topology: An Introduction.

